# Nitrogen Fixation and Anoxygenic Photosynthesis in Filamentous Non-Heterocystous Cyanobacterium of the Genus *Sodalinema* Isolated from Soda Lake

**DOI:** 10.3390/plants14233558

**Published:** 2025-11-21

**Authors:** Anastasia I. Kosyakova, Igor I. Rusanov, Tatiana P. Tourova, Elena E. Zakharova, Dimitry Y. Sorokin, Nikolay V. Pimenov, Olga S. Burakova

**Affiliations:** Winogradsky Institute of Microbiology, Research Center of Biotechnology, Russian Academy of Sciences, 60 Let Oktjabrja Pr-t, 7-2, Moscow 117312, Russia

**Keywords:** cyanobacteria, *Sodalinema*, soda lakes, nitrogen fixation, anoxygenic photosynthesis, sulfide

## Abstract

Saline and highly alkaline soda lakes are often characterized by a persistent nitrogen loss and high sulfide levels. Cyanobacteria are key aerobic diazotrophs in soda lakes, where light-dependent nitrogen fixation (NF) is crucial for sustaining ecosystem functioning. While sulfide is a well-known inhibitor of oxygenic photosynthesis, some cyanobacteria may tolerate it and utilize it via anoxygenic photosynthesis. In this study, we investigated the NF and anoxygenic photosynthesis in the genus *Sodalinema*, including non-heterocystous cyanobacteria widely distributed in soda and saline environments around the world and possessing an anaerobe-like nitrogenase. Our data suggest that their *nif*-operon could have been more likely acquired in soda or saline–alkaline lakes from natronophilic sulfate-reducing bacteria of the family *Desulfonatronovibrionaceae* than in the marine environment. It was shown that *Sodalinema* sp. P-1104, isolated from a southwestern Siberian soda lake, is capable of NF only in a light/dark switching mode, both in oxic and anoxic conditions. Sulfide did not suppress photosynthesis and stimulated NF up to threefold in oxygenic conditions. Anaerobic NF was obligately sulfide-dependent and supported by anoxygenic photosynthesis. However, removal of photosynthetic oxygen due to the high reducing potential of sulfide stimulated NF to a greater extent than does the use of sulfide through anoxygenic photosynthesis.

## 1. Introduction

Nitrogen fixation (NF) plays a significant role in the productivity of aquatic ecosystems, especially in nitrogen-deficient habitats [1,2]. This process is especially important in soda lakes, where continuous nitrogen loss occurs through denitrification, ammonia, and methylamines volatilization under highly alkaline conditions. In such environments, biological NF represents the main source of bioavailable nitrogen [3].

Among microorganisms capable of NF, cyanobacteria are major aerobic diazotrophs across diverse habitats. However, the activity of their nitrogenase enzyme complex is highly sensitive to oxygen, which irreversibly inactivates it. This presents a fundamental physiological challenge for cyanobacteria that produce oxygen during oxygenic photosynthesis.

To overcome this limitation, cyanobacteria have evolved different protective strategies for nitrogenase. Heterocystous cyanobacteria spatially separate NF and oxygenic photosynthesis by the formation of specialized nitrogen-fixing cells—heterocysts. Non-heterocystous cyanobacteria employ a combination of physical and physiological strategies to protect nitrogenase from oxygen. Physical protection involves the synthesis of exopolysaccharide (EPS) matrices and hopanoid lipids, both of which decrease the permeability of cell envelopes to molecular oxygen [4]. At the community level, they can form microaerobic or anaerobic niches within dense colonies and microbial mats, creating local environments favorable for NF [5].

Physiological mechanisms include the temporal separation of oxygenic photosynthesis and nitrogen fixation (with NF occurring primarily in the dark), and the activation of internal metabolic pathways that consume or reduce the production of oxygen. For example, photorespiration, photoreduction of O_2_ in the Mehler reaction, synthesis of antioxidant enzymes, replacement of typical D1 (PsbA) cofactors with atypical rD1 in the reaction center of PSII, etc. [6,7,8,9].

Another physiological mechanism facilitating NF in non-heterocystous cyanobacteria is the ability of some species to perform sulfide-dependent anoxygenic photosynthesis. Sulfide (H_2_S or HS^−^) can affect cyanobacteria in several ways: (1) it may act as a toxin that inhibits photosystem II (PSII) and suppresses photosynthetic activity; (2) it may have no significant effect; (3) in sulfide-tolerant species, it can serve as an alternative electron donor for the photosynthetic electron transport chain—a process known as anoxygenic photosynthesis [10]. During anoxygenic photosynthesis, the enzyme sulfide:quinone oxidoreductase (SQR) oxidizes sulfide and reduces the plastoquinone (PQ) pool, thereby feeding electrons into the photosynthetic electron transport chain. Under sulfidic, nitrogen-depleted conditions, this pathway can supply nitrogenase with additional reducing equivalents (ferredoxin, Fd) and ATP without the production of oxygen [11,12]. Consequently, the effect of sulfide on NF may be either inhibitory or stimulatory, depending on the physiological features of the cyanobacterium and the environmental conditions. However, information on the influence of sulfide on NF remains extremely limited. Experimental data exist for only a few cyanobacterial strains, and in most cases, the evidence is indirect [11,13,14,15].

It is well established that a few cyanobacteria, including *Coleofasciculus chthonoplastes* and representatives of the genus *Sodalinema*, possess anaerobe-like (or “desulfo”-type) *nif-*operons obtained via inter-phylum horizontal gene transfer (HGT) from sulfate-reducing bacteria [16]. Phylogenetic reconstructions suggest that the ancestors of marine isolates of *C. chthonoplastes* and *Sodalinema stali* (former *Phormidium lacuna*) HE10JO obtained nitrogenase genes from a species related to representatives of the families *Desulfovibrionaceae* [17] or *Desulfomonilaceae* [18]. Both families are widely distributed in different types of environments, including estuarine and marine, but not in soda or saline–alkaline lakes [19,20]. Genus *Coleofasciculus* is widely known as an inhabitant of salt marshes, intertidal lagoons, hypersaline pools, and other pH-neutral habitats [21]. Thus, the transfer of the *nif* operon from marine sulfate reducers to the representatives of *Coleofasciculus* seems logical. But the genus *Sodalinema* is distributed mainly in inland soda, saline alkaline, and salt lakes, and is significantly less common in marginal sea environments [22]. Therefore, for *Sodalinema*, the fact of *nif*-genes transfer taking place in marine conditions seems less likely, although it is not absolutely impossible. In this regard, the question remains unclear whether the act of inter-phylum HGT of the *nif*-operon was a single event or multiple events, and where it occurred—in marine or inland conditions, or independently in both.

In soda lakes, where high sulfide and alkaline pH coexist [3], members of the genus *Sodalinema* represent a particularly interesting model to study the interaction between NF and anoxygenic photosynthesis. An inconsistent taxonomy has made it difficult to assign these observations confidently to the genus. Several strains that were previously described under other generic names have since been reassigned to *Sodalinema* based on phylogenetic and taxonomic analyses. Therefore, the *Microcoleus chthonoplastes* ‘strain 11’ is now *Sodalinema stali* CCY9619, *Candidatus* Phormidium alkaliphilum PBR-2020, and *Candidatus* Phormidium yuhuli AB48 are now also recognized as representatives of the genus *Sodalinema* [22,23,24,25,26].

To date, only one strain of the genus *Sodalinema* (*S. stali* CCY9619, originally isolated from a marine microbial mat) has been directly studied for nitrogenase activity. It was shown that it can fix nitrogen under strictly anaerobic conditions with the addition of the inhibitor of oxygenic photosynthesis 3-(3,4-dichlorophenyl)-1,1-dimethylurea (DCMU) under continuous light during a 24 h experiment [23]. No information is available on its ability to fix nitrogen under oxic conditions or in the absence of DCMU. For alkaliphilic strains PBR-2020 and AB48, only *nif* gene expression was demonstrated in culture, photobioreactor, and natural habitat [24,25,26], which indirectly indicates the possibility of NF in oxic conditions, but this hypothesis needs further investigation.

For *S. stali* CCY9619, evidence for anoxygenic photosynthesis has also been obtained. This strain was shown to perform both oxygenic and anoxygenic photosynthesis simultaneously, with thiosulfate rather than elemental sulfur as the product of sulfide oxidation. However, this strain was unable to grow anaerobically using anoxygenic photosynthesis alone [27,28]. Despite these insights, there are still no data on how sulfide affects NF under nitrogen-depleted conditions in *Sodalinema*.

The objective of this study was to ascertain the ability of *Sodalinema* sp. P-1104 isolated from a soda lake to fix atmospheric nitrogen and to investigate the efficiency and peculiarities of this process, specifically the impact of sulfide and its capacity for anoxygenic photosynthesis.

## 2. Results

### 2.1. Ecophysiological Properties of the Strain Sodalinema sp. P-1104

**Figure 1:**
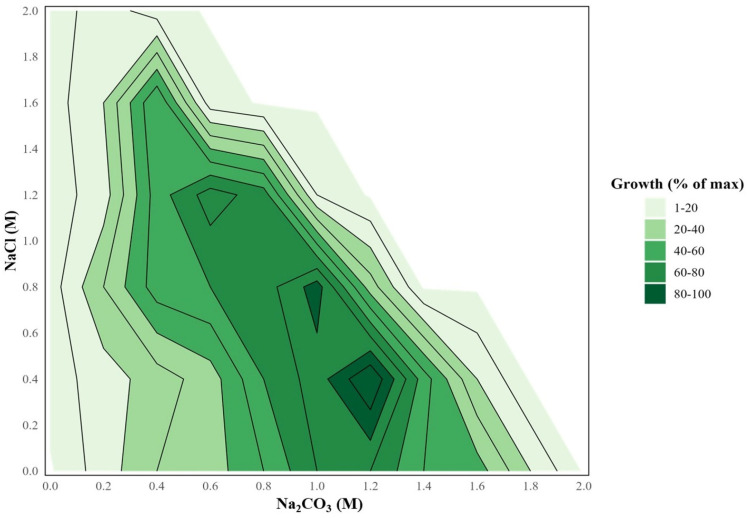
Growth of *Sodalinema* sp. P-1104 in the concentration matrix of NaCl and Na_2_CO_3_ at pH 10. The color gradient reflects biomass yield in mg of dry weight on the 7th day of cultivation. The white area reflects the ratio of salts at which complete dissolution did not occur (total Na^+^ > 4 M); therefore, growth was not measured in these variants.

Previous field observations showed that *Sodalinema* occurs in Lake Petukhovskoe in a range of total salinity from 30 to 200 g/L [22], indicating its wide range of salt tolerance. Indeed, growth experiments demonstrated that *Sodalinema* sp. P-1104 tolerated a wide range of salinity from 0.4 to 3.6 M total Na^+^ with an optimum at 2.8 M total Na^+^ (1.2 M Na_2_CO_3_ + 0.4 M NaCl; Figure 1). In the absence of chlorides at pH 10 (biomass yield at 0 M NaCl in Figure 1), the strain growth was observed in the range of 0.2 to 1.8 M Na_2_CO_3_ with an optimum at 1.0–1.2 M. Although sodium chloride can be used to compensate for the osmotic pressure of the brine when the carbonate concentration in the medium decreases, the strain is unable to grow with sodium chloride fully replacing sodium carbonate, even when the pH is maintained at 10 (biomass yield at 0 M Na_2_CO_3_ in Figure 1). Thus, the strain was qualified as a true NaCl-independent natronophile.

Among the media commonly used for cultivating cyanobacteria from highly mineralized soda lakes, medium M aligns most closely with the optimal salinity conditions estimated for strain P-1104. It also corresponds to the natural conditions in which *Sodalinema* is widely found in diazotrophic phototrophic communities in Kulunda steppe soda lakes [29]. Thus, we conducted further work using medium M containing 1 M of total carbonates at pH 10 (1.76 M of total Na^+^).

No diazotrophic growth was observed when incubated in liquid nitrogen-free medium M_-N_ under any lighting regimes (natural light, continuous light, or light/dark mode) and various incubation methods (with and without stirring). The experiments were repeated several times, each time without success. Bleaching of the cultures, observed as a yellowish discoloration visible to the naked eye, indicated nitrogen starvation.

### 2.2. Nitrogenase Gene Cluster

The genome of *Sodalinema* sp. P-1104 contains only one (incomplete) *nif*-operon located in the contig ending with the *nif*E gene (GenBank accession number NZ_SMDP01000020.1). Although the *nif*NB genes are absent from the available genome sequence, there is no reason to doubt their actual presence in the genome of this strain. The absence or damage of these genes would result in the absence of diazotrophic activity, but this did not happen (see below). Thus, the incompleteness of the operon does not interfere with further analysis.

**Figure 2:**
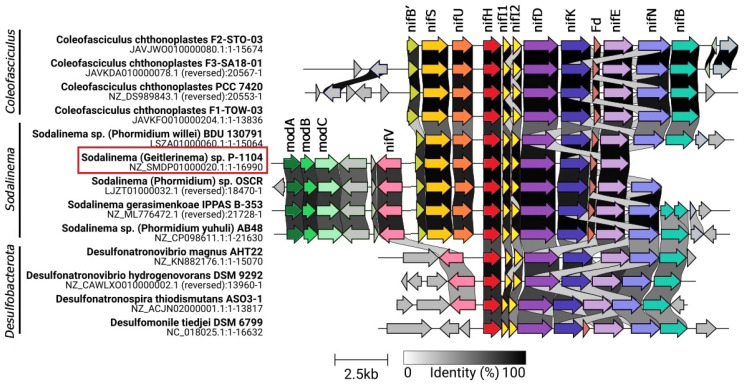
Gene synteny of nitrogenase metabolism in representative genomes of *Sodalinema* and *Coleofasciculus* (phyl. *Cyanobacteriota*) and families *Desulfovibrionia* and *Desulfomonilia* (phyl. *Desulfobacterota*). Genes associated with the *nif*-operon are highlighted in colors, and unknown genes are shown in gray. Abbreviations: *mod*A (dark green arrow), molybdenum ABC transporter, substrate-binding protein ModA; *mod*B (green), molybdenum ABC transporter permease protein ModB; *mod*C (light green arrow), molybdenum ABC transporter ATP-binding protein ModC; *nif*V (pink), homocitrate synthase (EC 2.3.3.14); *nif*S (yellow arrow), cysteine desulfurase (EC 2.8.1.7); *nif*U (orange arrow), iron–sulfur cluster assembly scaffold protein NifU; *nif*H (red arrow), nitrogenase (molybdenum–iron) reductase and maturation protein NifH; *nif*I (pale yellow arrow), nitrogen regulatory protein P-II, nitrogen-fixation associated; *nif*DK (purple and blue arrow), nitrogenase (molybdenum–iron) alpha and beta subunits; Fd (brown arrow), ferredoxin, 2Fe-2S; *nif*E (lilac arrow), nitrogenase FeMo-cofactor scaffold and assembly protein NifE; *nif*N (violet arrow), nitrogenase FeMo-cofactor scaffold and assembly protein NifN; *nif*B (turquoise arrow), nitrogenase FeMo-cofactor synthesis FeS core scaffold and assembly protein NifB. The studied strain is marked in the red frame.

Nitrogenase operon of *Sodalinema* sp. P-1104 has a structure *nif*VB’SUHDKE, identical to that previously found in *Coleofasciculus chthonoplastes* PCC 7420 [17] and other *Sodalinema* strains from marine and soda lake environments [18,26], which, in turn, are highly similar to those present in anaerobic bacteria of the phylum *Desulfobacterota*, namely the сlasses *Desulfovibrionia* and *Desulfomonilia* (Figure 2). It contains additional genes *nif*I1,2 encoding regulatory proteins P-II (or NifI1) and P-II’ (or NifI2), which are known to inhibit nitrogenase activity in the presence of ammonium in anaerobes [30], while in aerobic diazotrophs, these genes are usually absent [31]. The *nif* gene cluster of *Sodalinema* strains also contains flanking *nif*ENB genes encoding cofactor assembly proteins involved in the biosynthesis of Fe-Mo cofactors typical for *Desulfobacterota* instead of the *nif*ENXW genes typical for most cyanobacteria. On the opposite side, the typical cyanobacterial genes *ni*fB’SU are preserved, although the *nif*B’ gene is truncated and might not be functional (Figure 2).

**Figure 3:**
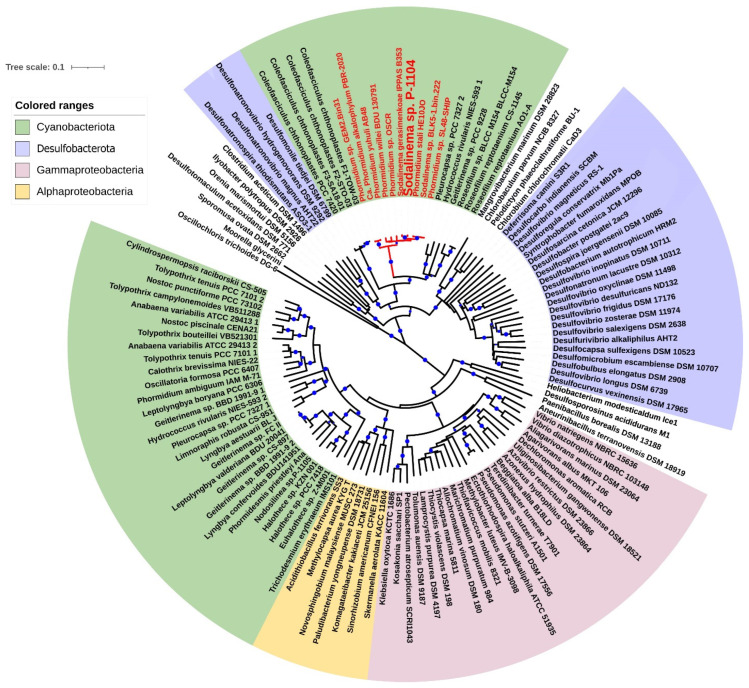
Phylogenetic tree based on the analysis of translated amino acid sequences of the *nifHDK* genes. This analysis involved 121 amino acid sequences. All ambiguous positions were removed for each sequence pair (pairwise deletion option). There were a total of 1252 positions in the final dataset. The optimal tree based on 100 bootstrap replicates was shown. Blue dots indicate bootstrap support levels > 80%. The scale shows 1 substitution per 10 amino acid residues. The names of *Sodalinema* strains are marked by red boldface.

The phylogenetic tree constructed through the analysis of the amino acid sequences of nitrogenase structural proteins NifHDK indicates that strains of *Sodalinema* spp. and C. *chthonoplastes* belong to a distinct subcluster among members of *Desulfobacterota*. This subcluster is peripherally associated with another subcluster comprising lithotrophic obligately natronophilic sulfate-reducing bacteria, *Desulfonatronospira thiodismutans*, *Desulfonatronovibrio magnus*, and *Desulfonatronovibrio hydrogenovorans* (fam. *Desulfonatronovibrionaceae*), inhabiting soda lakes worldwide [3] and *Desulfomonile tiedjei* DSM 6799 (fam. *Desulfomonilaceae*), isolated from sewage sludge [32] (Figure 3).

### 2.3. Determining the Ability to Fix Nitrogen in Different Regimes of Cultivation

**Figure 4:**
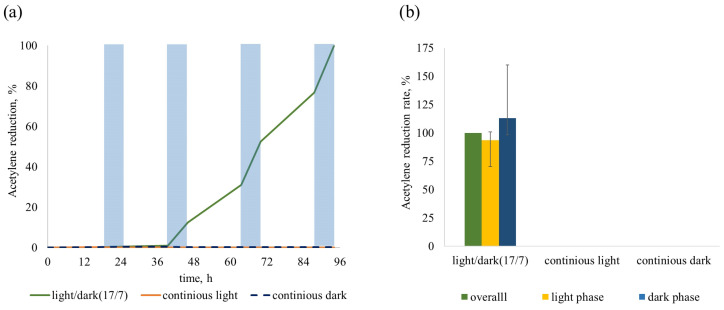
Generalized graphs of the AR by *Sodalinema* sp. P-1104 in different light regimes. (**a**) The dynamics of AR (shaded areas indicate the dark phases of incubation in the light/dark regime); (**b**) the ratio of ARRs calculated for the 3rd day of cultivation. The rates of AR in the light/dark regime of cultivation were taken as control and normalized to 100%. The graphs summarize the results of three independent experiments.

We examined the ability of *Sodalinema* sp. P-1104 to NF in conditions without blocking oxygenic photosynthesis under three light regimes: continuous light, continuous darkness, and alternating light and dark phases (17 and 7 h, respectively). The process occurred both during dark and light phases under a light/dark regime, although the rates were higher during dark phases. The strain did not show AR activity both under continuous light and continuous darkness (Table 1). The absence of AR in continuous darkness indicates that dark metabolism pathways, such as respiration or fermentation of endogenous compounds cannot support NF in this strain. Thus, *Sodalinema* sp. P-1104 was able to perform AR only in a light/dark regime of cultivation (Table 1). This was proven in different independent experiments, as shown in Figure 4. We observed a gradual increase in AR rate with daily measurements (Table 1). In most experiments, AR became detectable at the end of the second day (Figure 4a). Thus, all subsequent experiments were conducted under a light/dark regime; when it was necessary to estimate and compare the AR rates, we used data for the third day of incubation.

Although the gas phase was initially purged with argon to establish anoxic conditions, oxygen was produced during the light phases of incubation due to photosynthetic activity of the cyanobacterium; therefore, we consider light conditions oxic. It should be noted that biofilm formation proved to be crucial for AR activity. In experiments conducted with continuous stirring, AR was entirely absent under all lighting conditions (data not shown). We suggest that *Sodalinema* autonomously create local microaerobic or anaerobic microenvironments favorable for nitrogenase activity. Although the degree of aggregation could not be quantified, we observed visible sedimentation of the culture without biofilm formation. Throughout the incubation period, the cells remained easily resuspended by gentle shaking, indicating that the aggregates were loose and did not develop into stable biofilms. Consequently, all AR experiments presented in this study were performed without stirring, allowing the culture to aggregate. This likely reflects the natural ecological behavior of *Sodalinema*, which is predominantly found in benthic communities. Thus, we cannot consider AR activity in such conditions truly aerobic and henceforth define it as ”AR in oxic conditions”.

### 2.4. Effect of DCMU and Sulfide Addition on NF

Historically, studies on anaerobic NF in non-heterocystous cyanobacteria—such as those by [33]—used DCMU to inhibit oxygen production and maintain anoxia. To align our findings with the existing literature and investigate the role of anoxygenic photosynthesis in NF, we used experimental variants supplemented with DCMU, sulfide, or a combination of both.

**Figure 5:**
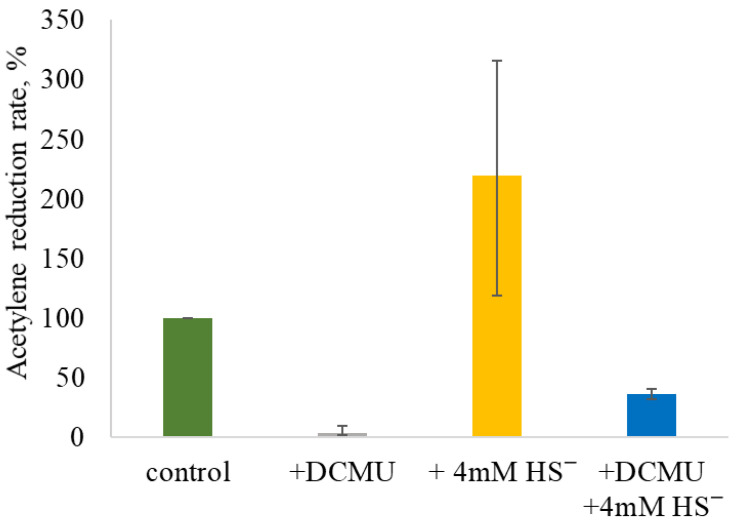
Summary plot of the effect of DCMU and/or sulfide addition on AR by *Sodalinema* sp. P-1104. The “control” corresponds to the experimental conditions without the addition of DCMU and/or sulfide and is taken as 100%. The graph summarizes the results of three independent experiments.

The 24 h ARR in the control variant incubated in a light/dark regime without DCMU and/or sulfide was 0.44 ± 0.27 µmol C_2_H_4_·g^−1^ dry weight·h^−1^. The addition of 7 µM DCMU almost completely inhibited AR (0.02 ± 0.02 µmol C_2_H_4_·g^−1^ dry weight·h^−1^). The addition of 4 mM sulfide stimulated AR by up to threefold compared to the control, reaching 0.99 ± 0.71 µmol C_2_H_4_·g^−1^ dry weight·h^−1^. The absence of NF inhibition suggests that 4 mM sulfide is not toxic to the strain under alkaline conditions. The addition of sulfide to DCMU-inhibited culture initiated AR activity, although the ARR was significantly lower compared to the control (0.24 ± 0.06 µmol C_2_H_4_·g^−1^ dry weight·h^−1^). These patterns were confirmed in three separate experimental series, and the summary plot is shown in Figure 5.

Thus, this series of experiments indicates the ability of *Sodalinema* sp. P-1104 to carry out NF in the presence of sulfide. The AR activity in samples with simultaneous addition of sulfide and DCMU suggests that the addition of sulfide allows obtaining reducing equivalents from another process than oxygenic photosynthesis, most likely via anoxygenic photosynthesis. To be able to carry out anoxygenic photosynthesis, the presence of the enzyme sulfide–quinone reductase (SQR, EC 1.8.5.4) is required.

### 2.5. Sulfide–Quinone Reductase

**Figure 6:**
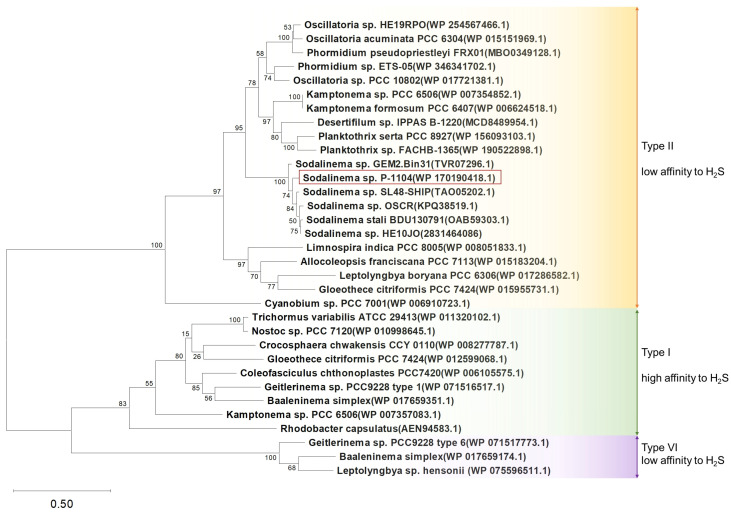
Maximum likelihood phylogenetic tree of SQR based on amino acid sequences of selected cyanobacteria. Bootstrap support values calculated from 1000; the tree scale represents the number of amino acid substitutions per site. Amino acid sequence of SQR of the anoxygenic purple bacterium *Rhodobacter capsulatus* was added to the tree due to its well-studied status. The studied strain is marked in the frame. The types of SQR were determined according to [34].

SQR is an enzyme that oxidizes sulfide and donates electrons to plastoquinone in the photosynthetic electron transport chain. Analysis of the genome of *Sodalinema* sp. P-1104 revealed only a single *sqr* gene. It belongs to a distinct subcluster within the type II cluster of SQRs (according to [34]) with an amino acid identity > 90% and with other SQRs belonging to the representatives of the genus *Sodalinema* and <75% with SQRs belonging to other cyanobacteria (Figure 6). Type II cluster was earlier characterized by low affinity to sulfide [34], meaning insensitivity to low concentrations and tolerance to higher concentrations of sulfide. The presence of SQR in the *Sodalinema* sp. P-1104 genome (GenBank accession number WP_170190418.1) confirms its genetic potential for sulfide-dependent anoxygenic photosynthesis.

It is important to note that in the genomes of *Sodalinema gerasimenkoae* IPPAS B-353 and *Sodalinema ‘yuhuli’* AB48, the *sqr* gene is missing. This suggests that these species lack the protein, or the gene could be located in a missing part of the genomes, although this possibility is unlikely given the high-quality assemblies of these genomes. Based on these findings, we hypothesize that these strains may have lost the *sqr* gene over the course of evolution, or they did not acquire it from an SQR-positive ancestor.

### 2.6. Anoxygenic Photosynthesis

**Figure 7:**
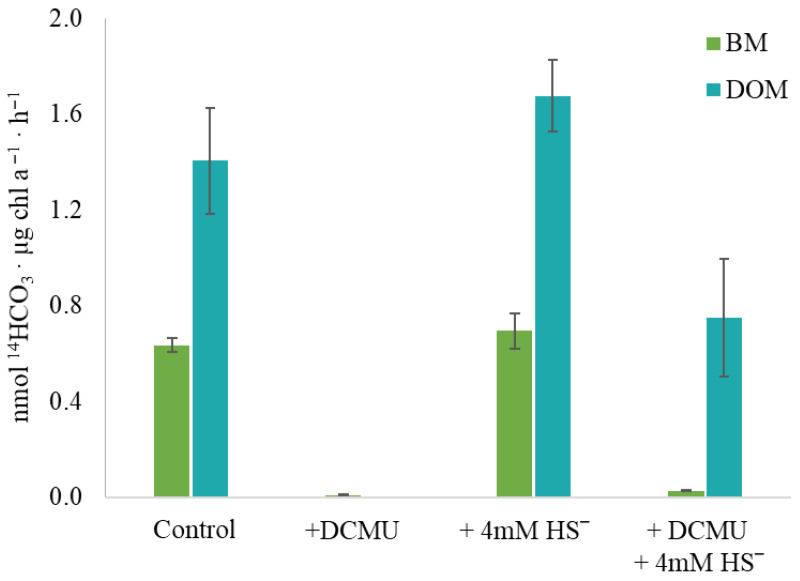
The effect of DCMU and/or sulfide on H^14^CO_3_^−^ assimilation by *Sodalinema* sp. P-1104. The “control” corresponds to the experimental conditions without the addition of DCMU and/or sulfide. BM—biomass, and DOM—dissolved organic matter. The culture was pre-incubated with sulfide prior to the experiment.

To determine the ability of *Sodalinema* sp. P-1104 to perform anoxygenic photosynthesis, the method of labeled carbon photoassimilation was used. Under experimental conditions that ensure oxygenic photosynthesis (control variant), the overall rate of H^14^CO_3_^−^ assimilation was 2.04 ± 0.19 nmol ^14^HCO_3_·µg chl *a*^−1^·h^−1^ with 31 ± 1.5% of labeled carbon included in the BM and 69 ± 10.5% included in the DOM. The addition of 4 mM sulfide did not affect (neither inhibit nor stimulate) the overall assimilation of H^14^CO_3_^−^ (2.37 ± 0.22 nmol ^14^HCO_3_·µg chl *a*^−1^·h^−1^) and did not change the ratio of ^14^C included in BM and DOM (29 ± 3% and 70 ± 6.3%, respectively) (Figure 7). As anticipated, the addition of DCMU nearly completely inhibited the assimilation of H^14^CO_3_^−^. With the combined addition of sulfide and DCMU, the overall rate of assimilation was 0.78 ± 0.25 nmol ^14^HCO_3_·µg chl *a*^−1^·h^−1^, which is about three times lower than in the control, and the proportion of H^14^CO_3_^−^ included in BM significantly decreased (to 3.8 ± 0%). Thus, the addition of sulfide to the culture inhibited by DCMU initiated the process of H^14^CO_3_^−^ assimilation, with its inclusion mainly in the DOМ.

### 2.7. Biologically Mediated and Physiological Sulfide Oxidation

To compare the extent of biologically mediated (via oxygen released during oxygenic photosynthesis) and physiological (via SQR) sulfide oxidation, an experiment was conducted to analyze sulfide consumption dynamics under various conditions over a 12 h period. Abiotic (chemical) sulfide oxidation was assessed by measuring sulfide loss in chemical controls with sterile medium (both in light and dark conditions). Biologically mediated oxidation was evaluated in samples incubated with cyanobacterial biomass under light, without DCMU addition. Physiological oxidation was determined by the loss of sulfide in samples with cyanobacterial biomass and DCMU. In the dark variants with cyanobacterial biomass, both chemical and physiological oxidation of sulfide could occur.

**Figure 8:**
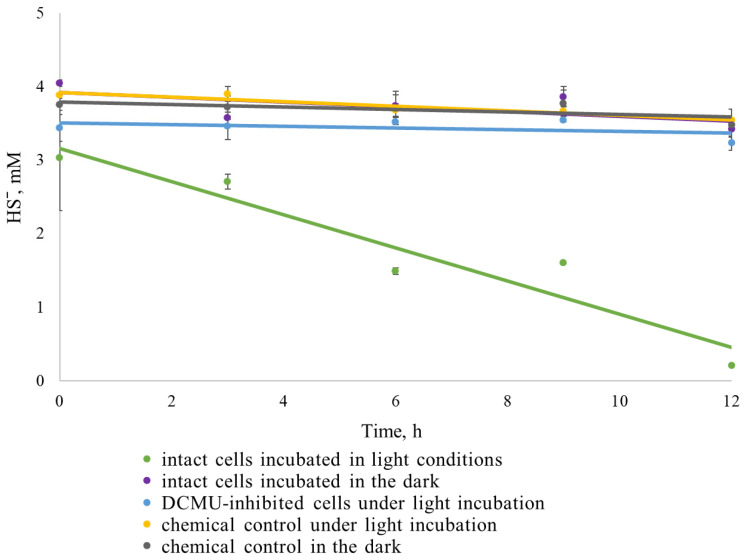
Dynamics of sulfide consumption by *Sodalinema* sp. P-1104 over 12 h under selected experimental conditions. Solid lines represent trends in sulfide concentrations over time. “Intact cells” refer to cultures incubated with sulfide under light or dark conditions to assess biologically mediated and physiological sulfide oxidation. “DCMU-inhibited cells” indicate samples with simultaneous addition of sulfide and DCMU to evaluate only physiological (SQR-dependent) oxidation. Chemical controls consisted of sterile medium with added sulfide to measure abiotic sulfide oxidation under both light and dark conditions.

A significant decrease in sulfide concentration was only detected in the light-exposed vials without DCMU, where photosynthetic oxygen was actively released (Figure 8). In all treatments without oxygen production—namely, the chemical controls, culture exposed to light with DCMU, and culture in the dark without additives—sulfide concentrations remained essentially unchanged, with no statistically significant differences observed between these treatments. Thus, under laboratory experimental conditions with millimolar concentrations of sulfide introduced, biologically mediated oxidation significantly exceeds physiological oxidation through SQR, which requires micromolar concentrations of sulfide [35]. Thus, the reduction of oxygen by sulfide may be an important factor in providing anoxic conditions and stimulating NF simultaneously with oxygenic photosynthesis.

### 2.8. The Effect of Sulfide Concentration on NF

To assess the effect of sulfide concentration on NF in *Sodalinema* sp. P-1104, we examined AR activity with sulfide ranging from 2 to 20 mM, both with and without DCMU addition. No inhibition of the AR process was observed at any sulfide concentration, regardless of DCMU addition.

**Figure 9:**
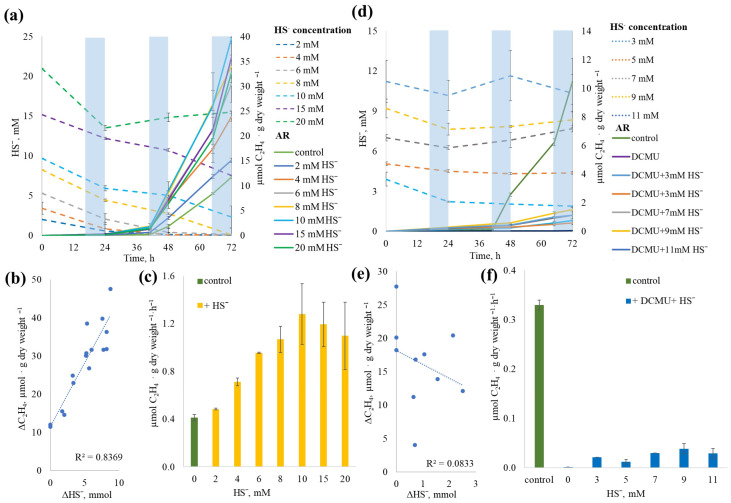
Effect of different sulfide concentrations on acetylene reduction (AR) by *Sodalinema* sp. P-1104 without (**a**–**c**) and with (**d**–**f**) addition of DCMU. (**a**,**d**) Solid lines show ethylene dynamics, and dashed lines show sulfide concentration dynamics; shaded blue areas indicate the dark phases of incubation in the light/dark regime. (**b**,**e**) Graphs show the correlation between sulfide consumption (∆HS^−^) and ethylene production (∆C_2_H_4_) over the entire duration of the experiments without and with DCMU addition, respectively. (**c**,**f**) ARRs at different sulfide concentrations (for the third day of incubation); green columns indicate ARR in intact cells without additions (control), and yellow and blue columns—ARRs in flasks with sulfide addition to intact and DCMU-inhibited cells, respectively.

In DCMU-free variants, sulfide consumption began on the first day of incubation. At concentrations between 2 and 8 mM, sulfide was completely consumed within 72 h, while at higher concentrations, residual sulfide remained (Figure 9a). A strong positive correlation (r = 0.91) was found between sulfide consumption and ethylene production (Figure 9b). A consistent increase in AR activity was observed up to 10 mM sulfide, with a near-linear relationship up to 8 mM; beyond this, AR rates plateaued (Figure 9c). Overall, sulfide addition stimulated AR activity by up to four times (from 0.44 to 1.53 µmol C_2_H_4_·g^−1^ dry weight·h^−1^).

In DCMU-treated samples, AR activity was significantly lower than in DCMU-free control (Figure 9d,e). Despite the addition of sulfide-initiated AR, its rate did not correlate with sulfide concentration (r = −0.29) (Figure 9f). Furthermore, sulfide concentration remained largely unchanged throughout the experiment in DCMU-treated variants. This lack of correlation is most likely due to the use of comparatively high sulfide concentrations, at which possible physiological oxidation or consumption remained within the analytical error of sulfide measurement. Consequently, subtle physiological changes could not be resolved against the high background levels.

## 3. Discussion

### 3.1. Nitrogen Fixation Among Cyanobacteria with “Desulfo”-Type of nif Genes

Information on NF in cyanobacteria possessing the same type of *nif* operon as *Sodalinema* remains rather limited and fragmented. Table 2 summarizes the available information on the evidence for NF in different phylogenetically confirmed *Sodalinema* strains, including the data obtained in the present study, as well as two strains of the genus *Coleofasciculus* due to the similar structure of the *nif*-operon. Three NF-related criteria were considered: the presence of genes encoding nitrogenase, evidence of nitrogenase activity in vivo, and the ability to grow on nitrogen-free media.

### 3.2. Hypothesis on the Origin of the Nitrogenase Operon

Although the fact of inter-phylum HGT from sulfate-reducing bacteria to cyanobacteria has been discussed since 2010 [17], the question of under what environmental conditions this could have occurred has not yet been addressed. Our reconstruction of the NifHDK phylogeny suggests that the transfer of structural nitrogenase genes could have more likely occurred from natronophilic representatives of the family *Desulfonatronovibrionaceae* (*Desulfonatronovibrio, Desulfonatronospira*), i.e., in a soda or saline–alkaline lake. Ecological preferences and biogeography of *Sodalinema* are generally in favor of this assumption [22]. More broadly, it can be hypothesized that *Sodalinema* originally evolved in inland soda or saline alkaline lakes and then spread to marine habitats. It was suggested in [25] that neutrophilic strains of *Sodalinema* may have been initially adapted to high pH because they possess the same genes involved in adaptation to alkaline conditions as alkaliphilic *Sodalinema* sp. PBR-2020 (with the exception of the single phytoene/squalene synthetase gene). This finding is also consistent with our hypothesis, although it leaves open the possibility of the opposite interpretation.

Although *Coleofasciculus* is widely distributed in pH-neutral habitats [21], its ancestors may also have acquired the *nif*-operon in saline–alkaline environments. Our observations show that *Coleofasciculus* can coexist with *Sodalinema* in such types of lakes. It was proved, for example, in the saline–alkaline Lake Khilganta in Transbaikalia, Russia [36]. Although the genus *Sodalinema* has not yet been described, it was identified as *Phormidium/Geitlerinema* in this work. We also morphologically detected the co-occurrence of *Coleofasciculus* and *Sodalinema* in Vtoroe Zasechnoe and Solenoe lakes in the Kurgan region (Russia), both with a total salinity of about 70–80 g/L and a pH of about 9 (data not published; for general information on the lakes, see [37,38]).

Thus, our hypothesis explains the phylogenetic closeness of NifHDK sequences in *Desulfonatronovibrionaceae* and representatives of both genera, *Sodalinema* and *Coleofasciculus*. However, it requires further detailed verification. Additionally, the presented data do not allow us to answer the question of whether the inter-phylum HGT was a single event or multiple events, i.e., whether *Sodalinema* and *Coleofasciculus* acquired their *nif*-genes independently or sequentially (the first event was inter-phylum from *Desulfonatronovibrionaceae* to cyanobacteria, and the second event was intra-phylum from *Coleofaciculus* to *Sodalinema*, or vice versa).

### 3.3. Nitrogenase Activity

#### 3.3.1. Nitrogenase Activity in Oxic Conditions

AR activity, both in dark and light phases of incubation in overall aerobic conditions (Figure 4), suggests that strain P-1104 employs some strategy of protecting nitrogenase from molecular oxygen. The higher AR activity in the dark phase suggests that temporal separation of NF and oxygenic photosynthesis is important for this strain. Moreover, the presence of AR activity in the light phase implies that additional protective mechanisms are also involved. Both external (environmental) factors and internal (physiological) mechanisms may play a crucial role in enabling nitrogen fixation in this cyanobacterium.

Among the external factors influencing oxygen content in the medium are salinity, agitation, and dissolved sulfide. The solubility of oxygen in aqueous solutions depends on both the total content and the composition of dissolved salts, which significantly complicates the measurement of the actual oxygen content in multicomponent solutions. The main component of the medium M used in this study is Na_2_CO_3_. The solubility of oxygen in 1 M Na_2_CO_3_ (106 ppt) solution at 25 °C is three times lower than in distilled water [39], while in seawater (30 ppt) it is only 1.2 times lower [40]. Thus, *Sodalinema* strains maintained in media simulating seawater (ASN-III medium) and hypersaline soda brine (medium M) are, in fact, initially exposed to different oxygen content conditions. Sulfide may impact NF through changes in the physicochemical conditions of the medium, reducing oxygen content and redox potential, which is supported by the increase in AR rate with the addition of sulfide (Figure 5) and the strong correlation (r = 0.91) between sulfide consumption and ethylene production (Figure 9b) in our experiments. In soda lake environments with active microbial sulfidogenesis, this resource for oxygen neutralization seems practically limitless.

Physiological mechanisms that protect nitrogenase in non-heterocystous cyanobacteria, other than the temporal separation of oxygenic photosynthesis and NF, involve the internal consumption or reduction of oxygen produced during photosynthesis [6]. These mechanisms collectively serve to lower intracellular oxygen levels and minimize nitrogenase inactivation. For instance, oxygen can be metabolically consumed through photorespiration, in which the oxygenase activity of RuBisCO consumes intracellular O_2_. Another process, the Mehler reaction, enables photoreduction of O_2_ at photosystem I, functioning as a controlled “oxygen sink” that dissipates excess electrons [7]. The activity of various antioxidant enzymes, such as peroxidase, catalase, and superoxide dismutase, further protects the nitrogenase complex from reactive oxygen species [8]. In addition, cyanobacteria can modulate the structure of their photosynthetic apparatus by replacing the typical D1 (PsbA) protein in photosystem II with an atypical rD1 isoform, which exhibits reduced oxygen-evolving activity under stress conditions [9]. Together, these processes create a dynamic balance between photosynthetic oxygen production and its intracellular removal, allowing nitrogen fixation to proceed even in cells that perform oxygenic photosynthesis. In addition, some non-heterocystous cyanobacteria can perform sulfide-dependent anoxygenic photosynthesis. This process enables electron flow independent of oxygen evolution and helps sustain an anaerobic intracellular environment favorable for nitrogenase activity. In the present study, we focused on investigating this mechanism.

In summary, our findings indicate that high salinity and soluble carbonate alkalinity of the environment, removal of molecular oxygen via biologically mediated sulfide oxidation, and formation of biofilms and aggregates may promote light-dependent NF in the presence of oxygenic photosynthesis in *Sodalinema* sp. P-1104. Other physiological processes may also be important and require further study.

#### 3.3.2. Anoxygenic Photosynthesis and Sulfide-Dependent Anaerobic Nitrogenase Activity

Our experiments with *Sodalinema* sp. P-1104 demonstrated both a lack of inhibition of photosynthesis by sulfide (Figure 7 and Figure 9) and a stimulatory effect of sulfide on NF in the presence of DCMU (Figure 5). When PSII is inhibited by DCMU, the flow of electrons through the oxygenic photosynthetic electron transport chain is blocked, preventing the supply of energy required for nitrogenase activity. However, in the presence of DCMU, sulfide (an alternative electron donor), and the SQR enzyme, anoxygenic photosynthesis is activated, providing the necessary energy for nitrogenase. Thus, we conclude that anaerobic NF in DCMU-inhibited *Sodalinema* sp. P-1104 is sulfide-dependent and mediated by anoxygenic photosynthesis. The initiation of H^14^CO_3_ incorporation into DCMU-inhibited samples upon the addition of sulfide confirmed our hypothesis (Figure 7). The reasons for the significant change in the proportion between bicarbonate incorporation into biomass and dissolved organic matter (DOM) remain unclear. The preferential release of labeled carbon as DOM in these experimental conditions can be attributed to the limited energy yield of anoxygenic photosynthesis, which is insufficient for biomass synthesis. Another explanation could be the lack of oxygen under DCMU treatment conditions, since oxygen is essential for the oxidation of polyunsaturated fatty acids [41]. *Sodalinema* species have previously been shown to exhibit high polyunsaturated fatty acid content, up to 57% of the total [22], and *Sodalinema stali* CCY9619 was shown to be incapable of anaerobic growth on sulfide, despite its ability to perform anoxygenic photosynthesis [28]. In contrast, *Geitlerinema* sp. (*Oscillatoria limnetica*) PCC9228, which lacks polyunsaturated fatty acids, demonstrated this ability [41,42].

Comparison of sulfide loss rates under different conditions (Figure 8) shows that biologically mediated oxidation significantly exceeds physiological oxidation. However, this does not imply that physiological oxidation is absent. According to previous studies, SQR operates efficiently at micromolar sulfide concentrations [43]. In our experiment, the use of high sulfide concentration (4 mM) may have obscured the detection of physiological oxidation processes. Under alkaline conditions, the toxicity of sulfide is reduced due to the dissociation of H_2_S, which may contribute to the observed resilience. The reduced AR activity observed in the simultaneous presence of DCMU and sulfide (Figure 9f) suggests that physiological oxidation of sulfide cannot fully sustain NF without functional PSII. Although physiological oxidation was not directly detected, the presence of AR activity under these conditions indicates that anoxygenic photosynthesis occurred, which could only be supported through SQR-mediated sulfide oxidation. Overall, these results highlight the complex interplay between sulfide metabolism and NF in *Sodalinema* sp. P-1104. However, the precise mechanisms and stoichiometry involved remain unclear. Future studies should explore these mechanisms and their regulation to better understand the adaptive strategies of cyanobacteria in sulfide-rich alkaline environments.

### 3.4. Absence of Growth in Nitrogen-Free Medium

Despite the presence of *nif*-genes and detected nitrogenase activity, all studied *Sodalinema* strains were unable to grow in nitrogen-free media (Table 2). Although [18] suggested potential NF capability in *Sodalinema stali* HE10JO and *Sodalinema* sp. HE10DO based on a 2–3 fold increase in OD_750_ during 7-day maintenance in nitrogen-free medium, this conclusion does not seem convincing. Firstly, the growth was statistically insignificant for strain HE10JO, and secondly, “weak growth” could be explained by numerous factors, such as utilization of internal nitrogen reserves stored within the cells or the presence of trace amounts of nitrogen compounds in the medium, which could sustain limited growth, or the growth of heterotrophic bacterial satellites, which impacted the absorbance at 750 nm. Thirdly, successive subcultures on nitrogen-free media were not performed, and thus stable diazotrophic growth was not definitively demonstrated.

To date, growth on nitrogen-free media has not been convincingly demonstrated for any of the cyanobacteria that possess a single *nif*-operon of the “desulfo” type. Such data are either absent (*Geitlerinema* sp. PСС 9228, *Roseofilum* spp.) or directly indicate a lack of diazotrophic growth (strains of *Sodalinema* and *Coleofasciculus*). One of the reasons may be the anaerobe-like structure of the *nif*-operon, which lacks genes associated with the protection of nitrogenase from oxygen and directly affecting the activity of nitrogenase [44]. For example, the absence of *nifXW* genes, typically present in most of the cyanobacterial *nif*-operons, may have a significant impact. It was earlier shown for a non-heterocystous nitrogen-fixing cyanobacterium, *Leptolyngbya boriana*, that *ΔnifW* and *ΔnifXnafY* phenotypes were characterized by a very low nitrogenase activity (<10% of the wild type) and loss of diazotrophic growth ability [44]. Although the direct function of *nifX* and *nifW* is not precisely known, it has been estimated that these genes strongly correlate with aerobic metabolism [31] and are suggested to be involved in protecting the FeMo-cofactor of nitrogenase from oxygen damage [45,46]. However, this reason cannot be the only one, and other physiological and ecological factors may be crucial [47]. In any case, the reasons why some nitrogen-fixing cyanobacteria are unable to grow diazotrophically still remain poorly understood.

### 3.5. Ecological Significance

Soda lakes are inland saline lakes with stable pH values above 9 due to a high content of carbonate species (HCO_3_^−^ + CO_3_^2−^) as dominating dissolved anions, and low levels of dissolved alkaline earth metals (Ca^2+^, Mg^2+^) [48]. This ratio of ions, HCO_3_^−^ + CO_3_^2−^ >> Ca^2+^ + Mg^2+^, makes such lakes one of the most stable high pH environments on Earth [49]. They possess several characteristics that influence microbial element cycling. The absence of Ca^2+^ results in an excess of soluble phosphorus; high content of soluble inorganic carbon in particular, hydrocarbonate, favors autotrophic organisms; the presence of free sulfide predominantly in the less toxic ionic form HS^−^, along with the chemical stability of linear polymeric sulfur as polysulfides, facilitate a highly active sulfur cycle; and the predominance of ammonium nitrogen primarily as toxic, volatile ammonia, and methylamines, particularly thrimethylamine, in their non-ionized, toxic, and volatile forms results in nitrogen loss and limitation, necessitating biological NF to sustain microbial communities [3,50,51,52]. Considering these features, cyanobacteria and anoxygenic purple bacteria capable of diazotrophy, autotrophy, and sulfide-dependent photosynthesis should play a significant role in the overall functioning of soda lake ecosystems.

The capacity for light-dependent NF has previously been demonstrated for phototrophic communities of soda lakes across a wide range of total salinities, although the most efficient diazotrophs—heterocystous cyanobacteria—are limited by relatively low salinity [14,29,36,53]. Oremland [14] showed that non-heterocystous cyanobacteria contribute substantially to NF in the oxic zone of the stratified soda lake Mono (California) and that the addition of sulfide stimulated this process. Genus *Sodalinema* studied in the current work was earlier shown to dominate in “non-heterocystous” communities exhibiting light-dependent NF in a range of 55–100 g/L [29]. However, little is known about the physiological characteristics of NF in non-heterocystous natronophilic cyanobacteria and the effect of sulfide on their nitrogenase activity. Thus, our findings expand current understanding of these issues and contribute to the understanding of microbial nitrogen and sulfur cycling processes that underpin primary productivity in soda lake ecosystems. Our results indicate the ecological significance of light-dependent NF by non-heterocystous cyanobacteria during the seasonal development of phototrophic communities and highlight the potential role of *Sodalinema* in nitrogen supply in these extreme but highly productive environments.

## 4. Materials and Methods

### 4.1. Object of the Study

The object of this study was the cyanobacterium *Sodalinema* sp. strain P-1104 isolated previously from Lake Petukhovskoe (Kulunda steppe, Altai region, Russia). The lake is also known in English-language literature as Cock Soda Lake [51,54] and has the following coordinates: 52°6′20.52″N, 79°9′22.19″ E. This strain was initially classified as *Geitlerinema* [54] but later reassigned to the genus *Sodalinema* on the basis of a polyphasic approach [22]. As a result, some database entries may still refer to it as *Geitlerinema*.

### 4.2. Cultivation and Biomass Measurements

*Sodalinema* sp. P-1104 culture was maintained in a medium M, the main mineral composition of which resembles soda lake conditions (g/L): Na_2_CO_3_—79.5; NaHCO_3_—21.0; KCl—2.0; K_2_HPO_4_·3H_2_O—0.5; KNO_3_—2.0; Na_2_SO_4_—1.4; FeCl_3_—0.0003; and EDTA—0.001; 1 mL of the A5 trace elements solution of the following composition (g/L): H_3_BO_3_—2.86; MgCl_2_·4H_2_O—1.81; ZnSO_4_·7H_2_O—0.222; Na_2_MoO_4_·2H_2_O—0.39; CuSO_4_·5H_2_O—0.079; and Co(NO_3_)_2_·6H_2_O—0.0494. Final pH was 10–10.5. In total, this medium contains 1 M of carbonates and 1.76 M of Na^+^. Nitrogen-free medium M (M_-N_) had the same composition except for KNO_3_. All cultivations and subsequent experiments were carried out under artificial white lighting with an intensity of 55 µmol·m^−2^·s^−1^ measured by Spectrometer LI-COR 180 (LI-COR Environmental, Lincoln, NE, USA).

To determine which ecological group (haloalkaliphile or natronophile) the strain P-1104 might belong to, and to determine the optimal concentration and ratio of salts for physiological experiments, the growth in the Na_2_CO_3_ vs. NaCl concentration matrix was studied. The basal salts (all except for carbonates) were used as in medium M. Concentration of Na_2_CO_3_ ranged from 0 to 2 M with a step of 0.2 M, and concentration of NaCl ranged from 0 to 2 M with a step of 0.4 M. The pH value in all variants was brought to 10 (which is typical for soda lakes) by 1 M HCl or 1 M KOH. In total, the matrix included 51 variants with a Na^+^ range from 0 to 4 M. The Na^+^ content was taken as the sum of sodium in NaCl and Na_2_CO_3_. Sodium from other basal salts was not taken into account.

Chlorophyll *a* content was determined spectrophotometrically from extracts prepared in 90% (*v*/*v*) acetone. A culture aliquot was transferred to a plastic tube containing glass marbles, followed by the addition of acetone. The mixture was vortexed for 3 min to break up the cells and then incubated at 4 °C for 12–16 h to complete the extraction. After incubation, the sample was centrifuged at 10,000 rpm, and the resulting supernatant was analyzed using a UNICO 2100 spectrophotometer (Cole-Palmer, IL, USA), with 90% acetone as the blank. Chlorophyll *a* concentration was calculated using the equations given in the review [55].

To determine dry weight, biomass was collected in a 2 mL Eppendorf tube and centrifuged at 4000 rpm for 10 min using a Jouan B4i/BR4i centrifuge (Jouan, France). The supernatant was removed, and the biomass was dried at 105 °C for 24 h.

### 4.3. Preparation of Sodium Sulfide Stock Solutions and Sulfide Analysis

Fresh anoxic stock solutions of Na_2_S 9H_2_O (ChimMed, Moscow, Russia) were prepared immediately before each experiment in boiled distilled water and stored in serum bottles under an argon atmosphere. Afterward, an aliquot of the stock solution, calculated for the experiment, was added to the medium volume used in the experiment, and the resulting sulfide concentration was measured using the method described below. If necessary, the procedure was repeated until the desired sulfide concentration was achieved in the medium vial. Samples for sulfide analysis were fixed in a 10% Zn-acetate (*w*/*w*) water solution, and HS^−^ was determined colorimetrically with the methylene blue method according to [56] using UNICO 2100, Cole-Palmer spectrophotometer.

### 4.4. Anoxygenic ^14^HCO_3_^−^ Photoassimilation

The ability of *Sodalinema* sp. P-1104 to perform anoxygenic photosynthesis was determined by measuring the activity of H^14^CO_3_^−^ incorporation into biomass. *Sodalinema* sp. P-1104 was grown in a full nitrogen-containing medium M, and the cells were pre-incubated for one day with sulfide (4 mM) before the experiment. After pre-incubation, the cells were washed three times in a fresh medium M_+N_ by centrifugation at 4000 rpm for 10 min. This was performed in order to remove exometabolites (dissolved exopolysaccharides and other organic substances, which could adsorb the labeled substrate and distort the experimental results). Then, the dissolved oxygen was removed from the resulting suspension by vacuuming.

Then, 5 mL of prepared (washed and vacuumed) suspension containing 4.05 µg chl*a·*ml biomass^−1^ was introduced into 10 mL serum vials and sealed with rubber stoppers, and the gas phase was replaced with argon. To inhibit oxygenic release by photosystem II (PSII), DCMU was added to a final concentration of 7 µM. Sulfide was injected in a concentration of 4.5–5 mM. After 1 h pre-incubation in the dark, 10 µCi per sample of ^14^C Na-bicarbonate was added to the cell suspension and incubated under artificial light (55 µmol·m^−2^·s^−1^) for 1 h. Parallel samples incubated in the dark served as a control. The experimental vials were incubated in a horizontal position on a magnetic rocking platform to improve mixing and prevent possible self-shading. After incubation samples were fixed with 15 mL of 1 M HCl. A large amount of concentrated acid was needed to neutralize the carbonate buffer of the medium. This experiment was carried out with two technical replicates. Incorporation of labeled carbon (^14^C), both in the biomass (BM) and dissolved organic matter (DOM), was analyzed. The samples were filtered at 0.1 atm through nylon filters with a pore diameter of 0.2 μm and washed with a 3-fold volume of filtered, slightly acidified 10% solution of NaCl. The biomass-incorporated ^14^C was quantified with a liquid scintillation counter Packard TRI-CarbTR 2400 (Packard, Downers Grove, IL, USA). The ^14^C incorporation into the DOM fraction was measured in the filtrates after conversion into CO_2_ by potassium persulfate and captured into alkaline scintillation liquid.

The intensity of photoassimilation of labeled HCO_3_^−^ was calculated using the following formula:

(1) $$I=\frac{\left(r_{\mathrm{light}}-r_{\mathrm{dark}}\right)}{R}\cdot \frac{\left[{{\mathrm{HCO}}_{3}}^{-}\right]}{T\cdot \left[\mathrm{Chl}\;a\right]},$$

where I—intensity of ^14^C incorporation (nmol HCO_3_**·**µg chl *a*^−1^ h^−1^), [HCO_3_^−^]—HCO_3_^−^ concentration (nmol**·**L^−1^), R—radioactivity of injected labeled bicarbonate (impulses**·**minute^−1^), r_light_ and r_dark_—radioactivity of the formed photoassimilation products in the light and in the dark, respectively (impulses/minute), Т—time of incubation (hour), and [Chl *a*]—chlorophyll *a* content (µg chl *a***·**L^−1^).

For calculations, the bicarbonate concentration [HCO_3_^−^] in the medium М was determined by a two-step titration with 1 M HCl, using phenolphthalein and methyl orange indicators.

### 4.5. Nitrogenase Activity Experiments

Nitrogenase activity was determined by the acetylene reduction (AR) assay [57] using an approach specifically adapted for cyanobacteria ([33] with modifications).

#### 4.5.1. Preparation of Biomass

For all AR experiments, *Sodalinema* sp. P-1104 was grown in medium M with a reduced amount of nitrate (5.9 mM instead of 19.8 mM) under artificial light (55 µmol·m^−2^·s^−1^) with continuous stirring for 5 to 7 days. The resulting biomass was washed three times with a fresh nitrogen-free medium M (M_−N_). It was resuspended and incubated for 4 to 7 days at room temperature 22–25 °C under natural light (on the window) without shaking to deplete endogenous nitrogen stores. Long-time incubation under natural light conditions was chosen instead of 24–36 h of constant light on a shaker, according to [33]. In this case, visual bleaching was not observed, but the fact of phycobiliprotein degradation was confirmed spectrophotometrically according to [23]. For this analysis, nitrogen-starved intact cells were examined using a Cary 100 Bio UV–Visible spectrophotometer (Varian, USA). The absorption spectra showed a pronounced decrease in the phycocyanin peak at approximately 625 nm, indicating the degradation of light-harvesting pigments associated with nitrogen depletion.

#### 4.5.2. AR Assay

Aliquots of the prepared biomass were transferred into 50 mL serum vials containing 20 mL of medium, sealed with rubber stoppers, and flushed with argon. Acetylene was then injected into a final concentration of 3% (*v*/*v*) in the gas phase. Flasks were subsequently placed under the experimental conditions described in Section 4.5.3.

The amount of ethylene released in the gas phase was determined using a Crystal 2000 gas chromatograph (Chromatek, Russia) equipped with a flame ionization detector twice a day at the time of switching between the light and dark phases of incubation.

#### 4.5.3. Experimental Setup

To assess the ability of *Sodalinema* sp. P-1104 to fix nitrogen under various illumination regimes, we performed AR assays under three conditions: continuous light (55 µmol photons m^−2^ s^−1^), continuous darkness, and alternating light/dark phases (17 h light/7 h dark). The duration of the phases was set as close as possible to the natural intervals of 16/8 h. All further AR experiments were conducted only under a light/dark regime. The total duration of each experiment was 4 days (96 h). The temperature was maintained at 25 ± 1 °C. Each experiment included a dark control (continuous darkness) to evaluate potential NF activity driven by fermentation of endogenous carbon reserves.

In experiments testing the effects of sulfide or DCMU, the same biomass preparation and incubation protocols were applied. DCMU was added at a final concentration of 7 µM to inhibit oxygenic photosynthesis. Sulfide (2–20 mM) was injected into the vials at the start of incubation. Its concentrations were measured dynamically during the experiments, with samples collected at each light/dark transition.

#### 4.5.4. Data Processing and Normalization

All experiment variants were carried out in three independent repetitions (biological replicates), and each experiment was performed in two technical replicates. In each experiment, the amount of biomass used varied within the range of 40–50 mg dry weight, which was equivalent to 30–50 µg chl *a* per sample.

AR activity was calculated as µmol C_2_H_4_·g^−1^·dry weight·h^−1^. Because AR experiments with different light regimes and with the addition of DCMU and/or sulfide were conducted in three independent series, the results were normalized to the control variant (light/dark regime without any additives). The AR activity of each experimental treatment was expressed as a percentage of the control, set as 100%. Data variability is presented as the observed range (minimum–maximum) among replicate measurements.

### 4.6. Comparison of the Rate of Chemical and Biological Oxidation of Sulfide

To compare chemical, physiological (metabolic), and biologically mediated (i.e., indirectly via oxygen released during oxygenic photosynthesis) sulfide oxidation, 15 mL of medium M was introduced into 20 mL vials, and the gas phase was replaced with argon. Biomass of *Sodalinema* sp. P-1104 in concentration 45 µg chl *a* per sample, previously washed and resuspended in fresh medium M, was added together with sulfide at an initial concentration of 3–4 mM. To inhibit oxygenic photosynthesis, DCMU was introduced into selected vials at a concentration of 7 µM. The vials were incubated for 12 h under continuous illumination (experimental group) and in darkness (dark control). Additionally, two types of chemical controls were prepared: one under light and the other in darkness, both without biomass. Samples were taken every 3 h to measure sulfide concentrations. All experiments were conducted in duplicate.

### 4.7. Genome Analysis and Phylogenetic Tree Construction

The genome assembly of *Sodalinema* sp. P-1104 is available in the GenBank database under accession number GCF_012911965.1.

Evolutionary relationships of NifHDK from different bacterial taxa were inferred using the minimum evolution method [58]. This analysis involved 121 amino acid sequences. All ambiguous positions were removed for each sequence pair (pairwise deletion option). There were a total of 1252 positions in the final dataset. Evolutionary analyses were conducted in MEGA11 [59]. The optimal tree based on 100 bootstrap replicates was shown. Visualization of the tree was performed by online service iTOL v. 6.8 [60]. The neighborhood estimation and visualization of nitrogenase gene syntheses were performed using the CAGECAT release version 1.0 [61].

Protein sequences of the *sqr* gene-encoding sulfide–quinone reductase (SQR) were inferred from *Sodalinema* sp. P-1104 genome, other available genomes of *Sodalinema*, and other (mostly phototrophic) *sqr*-incoding bacterial genomes available in the GenBank, except for *Sodalinema* sp. HE10JO available from the IMG database. The dataset primarily consisted of cyanobacterial sequences, along with a *Rhodobacter capsulatus*, which is known for its well-characterized sulfide–quinone reductase [62]. Sequences were aligned using MUSCLE [63]. Phylogenetic analysis was performed using MEGA11 with the maximum likelihood (ML) method applying 1000 bootstrap replicates.

## 5. Conclusions

This study presents the first experimental data on NF and anoxygenic photosynthesis in a natronophilic cyanobacterium of the genus *Sodalinema*, isolated from extreme soda lake environments and possessing an anaerobe-like *nif*-operon (*nifVBSUHDKE* with regulatory *nifI1,2* genes). *Sodalinema* sp. P-1104 is able to perform NF only in a light/dark regime of cultivation, both in oxic and anoxic (DCMU-treated) conditions in a highly mineralized sodium carbonate-rich medium imitating natural soda lake brines. Anaerobic NF occurs only in the presence of sulfide and is supported by anoxygenic photosynthesis. While sulfide is often inhibitory for neutrophilic marine cyanobacteria, its addition to alkaliphilic *Sodalinema* sp. P-1104 remarkably stimulated NF up to threefold in oxic conditions. This stimulation is primarily due to the high reducing potential of sulfide and the removal of photosynthetic oxygen, rather than directly fueling NF via anoxygenic photosynthesis. These findings reveal crucial ecological adaptations of *Sodalinema* sp. P-1104, highlighting its physiological resilience and significant role in primary production and nutrient cycling within challenging polyextreme soda lake ecosystems.

## Figures and Tables

**Table 1:** The rates of acetylene reduction (ARR, µmol С_2_Н_4_·g dry weight^−1^·h^−1^) by *Sodalinema* sp. P-1104 under different light regimes during 4 days of measurements.

Day of Measurements	Light/Dark			Continuous	Continuous
	(17/7 h) Regime			Light	Darkness
	24 h	Light Phase ARR	Dark Phase ARR	24 h ARR	24 h ARR
	ARR				
1	0.02 ± 0.02	0	0.05 ± 0.05	0	0.02 ± 0.02
2	0.41 ± 0.14	0.02 ± 0.01	1.45 ± 0.09	0	0
3	1.37 ± 0.07	0.88 ± 0.11	2.7 ± 0.23	0	0
4	1.63 ± 0.11	1.14 ± 0.08	2.94 ± 0.15	0	0

**Table 2:** Evidence of nitrogen fixation by strains of the genera *Sodalinema* and *Coleofasciculus*.

Strains		Ecology (Geography)	Nitrogen Fixation Evidence			Refs.
Original Name	Phylogenetic Affiliation		Genetics	Nitrogenase Activity	Growth in Nitrogen-Free Medium	
*Microcoleus chthonoplastes* ‘strain 11’	*Sodalinema stali* CCY9619 (t.a. species, type strain)	Marine (microbial mats of Mellum, North Sea, Germany)	*nifH* gene belongs to “desulfo”-type	AR: Anaerobically with DCMU in the light during 24 h-long experiment	No	[22,23]
*Phormidium lacuna* HE10JO	*Sodalinema stali* HE10JO (t.a. species)	Marine (rockpools in Helgoland, North Sea, Germany)	*nif*-gene cluster *nifVBSUHDKENB*	n/d	Weak (2–3 fold increase in OD_750_ in 7 days both in light and light/dark regimes), statistically insignificant	[18]
*Phormidium lacuna* HE10DO	*Sodalinema* sp. HE10DO (ph.c.)	Marine (rockpools in Helgoland, North Sea, Germany)	n/d	n/d	Weak (about 2,5 fold increase in OD_750_ in 7 days both in light and light/dark regimes), statistically significant	[18]
*Phormidium yuhuli* AB48	*Sodalinema* sp. AB48 (ph.c.)	Saline alkaline (industrial photobioreactor environment initially designed to grow *Spirulina*, Canada)	*nif*-gene cluster *nifVBSUHDKENB*	Proteome: *nifBSUHDK*-genes expression in culture	No (under aerobic conditions with either continuous lighting or a 12 h light/dark regime)	[26]
*Сa.* “Phormidium alkaliphilum” PBR-2020	*Sodalinema* sp. PBR-2020 (ph.c.)	Soda lake, (Cariboo Plateau, Canada)	*nif*-gene cluster *nifVBSUHDKENB*	Proteome: *nifHDK*-genes expression in photobioreactor and natural environment	n/d	[24,25,26]
*Geitlerinema* sp. P-1104	*Sodalinema* sp. P-1104 (t.a. genus)	Soda lake Petukhovskoe (Kulunda steppe, Russia)	*nifH* gene belongs to “desulfo”-type; *nif*-gene cluster *nifVBSUHDKE*	AR: Only in the light/dark regime aerobically or anaerobically (with DCMU + sulfide or sulfide alone) during 96 h-long experiment	No (under aerobic conditions under natural light, continuous lighting and light/dark regime)	[22], this study
*Microcoleus chthonoplastes* PCC7420	*Coleofasciculus chthonoplastes* PCC7420 (t.a. species, type strain)	Marine (Sippewissett Salt Marsh, USA)	*nifH* gene belongs to “desulfo”-type; *nif*-gene cluster *nifBSUHDKENB*	AR: No (anaerobically with DCMU during 30 h-long experiment) qRT-PCR: No *nifHDK* genes expression in culture	No	[17]
*Microcoleus* sp. CCY0002	*Coleofasciculus* sp. CCY0002 (ph.c.)	Marine (Schiermonnikoog, North Sea, The Netherlands)	*nifHDK* genes belong to “desulfo”-type	AR: No (anaerobically with DCMU during 30 h-long experiment) qRT-PCR: No *nifHDK* genes expression in culture, but positive result on *nifH* gene expression in natural environment	No	[17]

Note: AR—acetylene reduction, DCMU—3-(3,4-dichlorophenyl)-1,1-dimethylurea, n/d—no data, ph.c.—phylogenetically confirmed (phylogenetic affiliation to the genus Sodalinema was shown without taxonomic description), qRT-PCR—quantitative reverse transcription-polymerase chain reaction; and t.a.—taxonomically approved (validly published under the Botanical Code (ICN) and valid under Bacteriological Code (ICNP)).

## Data Availability

All data generated or analyzed during this study are included in this published article. All data are available upon request to the corresponding authors.
